# The Effect of Low-Dose Proteasome Inhibition on Pre-Existing Atherosclerosis in LDL Receptor-Deficient Mice

**DOI:** 10.3390/ijms18040781

**Published:** 2017-04-07

**Authors:** Nicola Wilck, Mandy Fechner, Cristian Dan, Verena Stangl, Karl Stangl, Antje Ludwig

**Affiliations:** 1 Medizinische Klinik für Kardiologie und Angiologie, Charité–Universitätsmedizin Berlin, Campus Mitte, 10117 Berlin, Germany; m.fechner@medizin-im-gruenen.de (M.F.); cristian.dan@charite.de (C.D.); verena.stangl@charite.de (V.S.); karl.stangl@charite.de (K.S.); antje.ludwig@charite.de (A.L.); 2 Experimental and Clinical Research Center, a Joint Cooperation between the Charité-Universitätsmedizin Berlin and Max Delbrück Center for Molecular Medicine in the Helmholtz Association, 13125 Berlin, Germany; 3 DZHK, German Center for Cardiovascular Disease, Partner Site Berlin, 10115 Berlin, Germany

**Keywords:** atherosclerosis, proteasome inhibition, inflammation

## Abstract

Dysfunction of the ubiquitin-proteasome system (UPS) has been implicated in atherosclerosis development. However, the nature of UPS dysfunction has been proposed to be specific to certain stages of atherosclerosis development, which has implications for proteasome inhibition as a potential treatment option. Recently, low-dose proteasome inhibition with bortezomib has been shown to attenuate early atherosclerosis in low-density lipoprotein receptor-deficient (LDLR^−/−^) mice. The present study investigates the effect of low-dose proteasome inhibition with bortezomib on pre-existing advanced atherosclerosis in LDLR^−/−^ mice. We found that bortezomib treatment of LDLR^−/−^ mice with pre-existing atherosclerosis does not alter lesion burden. Additionally, macrophage infiltration of aortic root plaques, total plasma cholesterol levels, and pro-inflammatory serum markers were not influenced by bortezomib. However, plaques of bortezomib-treated mice exhibited larger necrotic core areas and a significant thinning of the fibrous cap, indicating a more unstable plaque phenotype. Taking recent studies on favorable effects of proteasome inhibition in early atherogenesis into consideration, our data support the hypothesis of stage-dependent effects of proteasome inhibition in atherosclerosis.

## 1. Introduction

Accumulating evidence suggests that the ubiquitin-proteasome system (UPS) plays an important role in atherogenesis. The UPS represents the major pathway for intracellular protein degradation and is involved in numerous atherosclerosis-relevant processes, such as inflammation and management of oxidative stress [1,2]. The 20S proteasome core complex contains three distinct proteolytic activities for hydrolysis of proteins into small peptides [3] and can be targeted by a variety of inhibitors [4]. Proteasome inhibitors have gained attention by exhibiting potent anti-inflammatory properties [5]. In addition, inhibition of the proteasome with low doses of inhibitors has been shown to have anti-oxidative effects in vitro and in vivo, without toxic side effects [6]. Consequently, proteasome inhibitors were attributed anti-atherogenic properties.

Recently, we showed that treatment of low-density lipoprotein receptor-deficient (LDLR^−/−^) mice with low doses of bortezomib attenuates early atherosclerotic lesion formation [7]. Beneficial effects from proteasome inhibition in early atherosclerosis are conceivable, since a dual role of the UPS in atherosclerotic plaque progression was postulated [1,8]. It was assumed that the UPS may be overreactive at an early stage, thereby driving the inflammatory response. With disease progression, vascular proteasomal activity may ultimately decrease [1,9], thus potentially narrowing the therapeutic window for proteasome inhibition. Moreover, a shift in proteasome composition, as described for cardiac disease [10], may influence the responsiveness to proteasome inhibition [11] in the course of atherosclerosis.

Recent studies investigating proteasome inhibition in different animal models of atherosclerosis have yielded conflicting results, ranging from pro-atherogenic effects [12,13] to anti-atherogenic effects [7,14]. Comparability of these studies is limited as they use different animal models, proteasome inhibitors, and doses, as well as target different stages of atherosclerosis. In addition to the above-mentioned atherosclerosis stage dependency, the effects of proteasome inhibitors may be particularly dose-dependent. We have previously shown that a six-week bortezomib treatment at a very low dose (50 μg/kg body weight (BW) ameliorates the establishment of early atherosclerosis in LDLR^−/−^ mice. Considering the suggested dual role of the UPS in atherosclerosis [1,2,8,9] and the deduced stage-dependent effects of proteasome inhibition therefrom, we asked whether the same treatment regimen is beneficial or detrimental in established advanced atherosclerosis in LDLR^−/−^ mice. Overall, investigation of the effect of bortezomib on advanced atherosclerosis is of clinical relevance, since bortezomib is currently being used in multiple myeloma patients [15]—an elderly patient cohort with an increased atherosclerosis prevalence.

Therefore, we investigated the effect of a six-week low-dose bortezomib treatment on pre-existing advanced atherosclerosis in Western-type diet-fed LDLR^−/−^ mice. Bortezomib treatment at this stage did not alter lesion burden, but influenced plaque composition by promoting features of plaque instability. Taking recent studies on favorable effects of bortezomib treatment in early atherogenesis in LDLR^−/−^ mice into consideration, the current data provides further evidence for a stage-dependent role of the UPS in atherosclerosis.

## 2. Results

### 2.1. Low-Dose Bortezomib Treatment Was Efficient and Well-Tolerated

**Figure 1:**
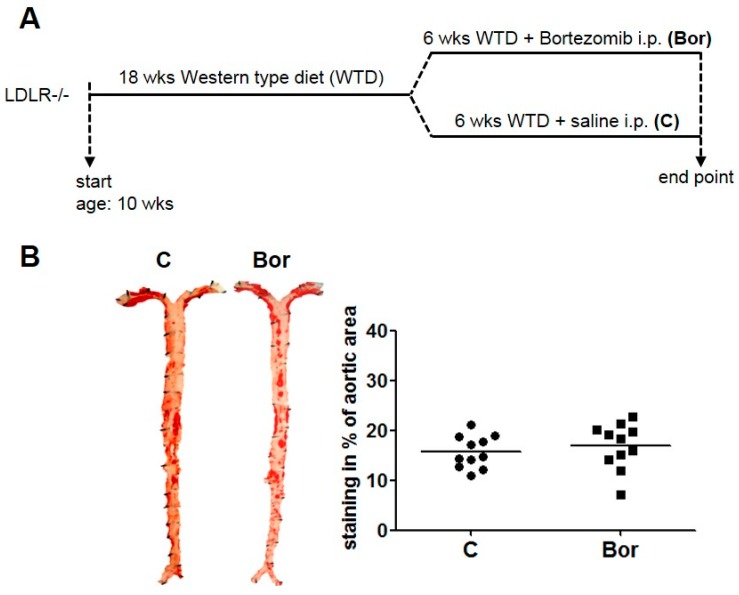
Atherosclerotic lesion burden in the aorta. (**A**) Experimental design. Male low-density lipoprotein receptor-deficient (LDLR^−/−^) mice were fed a Western diet for 24 weeks and were treated with intraperitoneal injection of saline (**C**) or bortezomib (Bor) during the last 6 weeks. (**B**) En face Oil Red O staining of Bor and C aortae revealed no differences in the atherosclerotic lesion area. Representative images and the quantification are shown. *n* = 11 per group.

To investigate the effect of low-dose proteasome inhibition on pre-existing atherosclerosis, male LDLR^−/−^ mice were fed a Western type diet for 18 weeks, starting from the age of 10 weeks. Western diet was then continued for another 6 weeks with intraperitoneal injections of bortezomib (Bor) or saline (C) (Figure 1A). Mice were injected with 50 μg/kg BW bortezomib twice weekly, a low dose previously shown to be non-toxic [7].

Treatment was well tolerated. Body weight was similar in both groups at the end of the treatment period (Table 1). Bor treatment had no influence on plasma total cholesterol or triglyceride levels (Table 1). Bor-treated mice showed a significant inhibition of the chymotrypsin-like proteasomal activity, as measured in liver lysates 24 h after the final injection (Table 1).

### 2.2. Bortezomib Had No Effect on Lesion Size and Macrophage Infiltration

**Figure 2:**
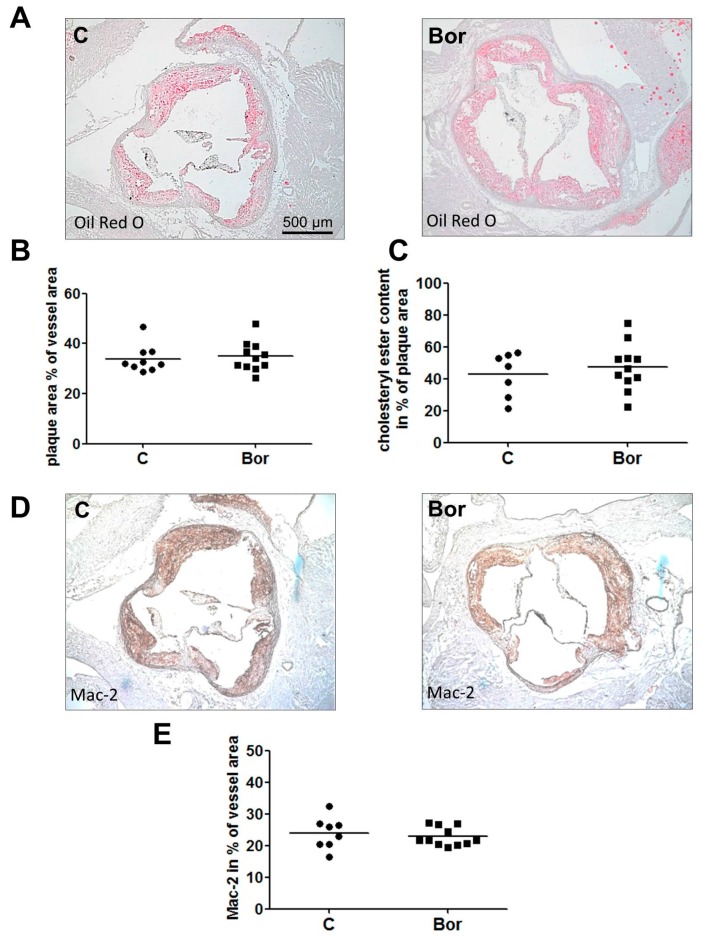
Atherosclerotic lesion size, lipid accumulation, and macrophage infiltration in the aortic root. Oil Red O staining of aortic root cryosections showed similar lesion sizes and similar cholesteryl ester content in Bor and C mice. (**A**) Representative Oil Red O aortic root sections. (**B**) Lesion area quantification. (**C**) Quantification of plaque cholesteryl ester content. Galectin-3 (Mac-2) staining of aortic root cryosections revealed similar macrophage content in Bor and C lesions. (**D**) Representative Mac-2 stainings. (**E**) Mac-2 quantification. *n* = 11 per group. Bor = bortezomib; C = saline control.

En face Oil Red O staining revealed marked atherosclerosis in both C and Bor mice, indicative of advanced atherosclerosis. Bor treatment had no effect on the en face lesion area compared to saline treatment (C: 15.74% ± 0.97%; Bor: 16.90% ± 1.39%; *p* = 0.502; Figure 1B). These results are consistent with lesion area quantification in the aortic root (C: 33.91% ± 1.86%; Bor: 34.86% ± 1.79%; *p* = 0.719; Figure 2A,B). Likewise, quantification of the Oil Red O positive plaque area yielded in similar results for both groups (C: 42.73% ± 5.24%; Bor: 47.33% ± 4.44%; *p* = 0.519; Figure 2C), indicating that lipid accumulation in atherosclerotic lesions of the aortic root is not influenced by Bor treatment.

**Figure 3:**
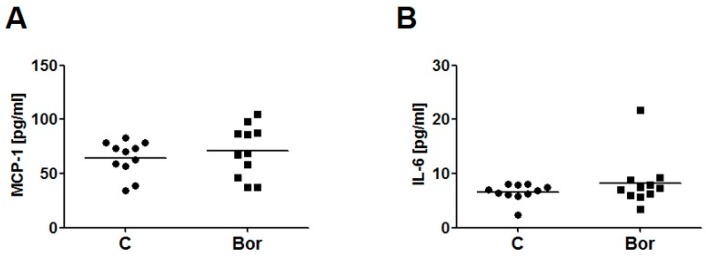
Serum levels of inflammatory markers monocyte chemoattractant protein 1 (MCP-1) and interleukin (IL)-6. Both monocyte chemoattractant protein-1 (**A**) and interleukin-6 (**B**) serum levels remained unaltered by Bor treatment. *n* = 11 per group. Bor = bortezomib; C = saline control.

For analysis of macrophage content in aortic root lesions, Galectin-3 (Mac-2) immunohistochemistry was performed. No differences in macrophage content between either group were detected (C: 23.97% ± 1.76%; Bor: 22.81% ± 0.89%; *p* = 0.710; Figure 2D,E). Correspondingly, serum levels of the pro-inflammatory chemokine monocyte chemoattractant protein 1 (MCP-1) (C: 64.44 ± 4.84 pg/mL; Bor: 70.75 ± 7.19 pg/mL; *p* = 0.475) as well as serum levels of interleukin (IL)-6 (C: 6.57 ± 0.48 pg/mL; Bor: 8.23 ± 1.43 pg/mL; *p* = 0.555) did not significantly differ between Bor and C mice (Figure 3A,B).

### 2.3. Bortezomib Influenced Plaque Composition Towards a Vulnerable Phenotype

**Figure 4:**
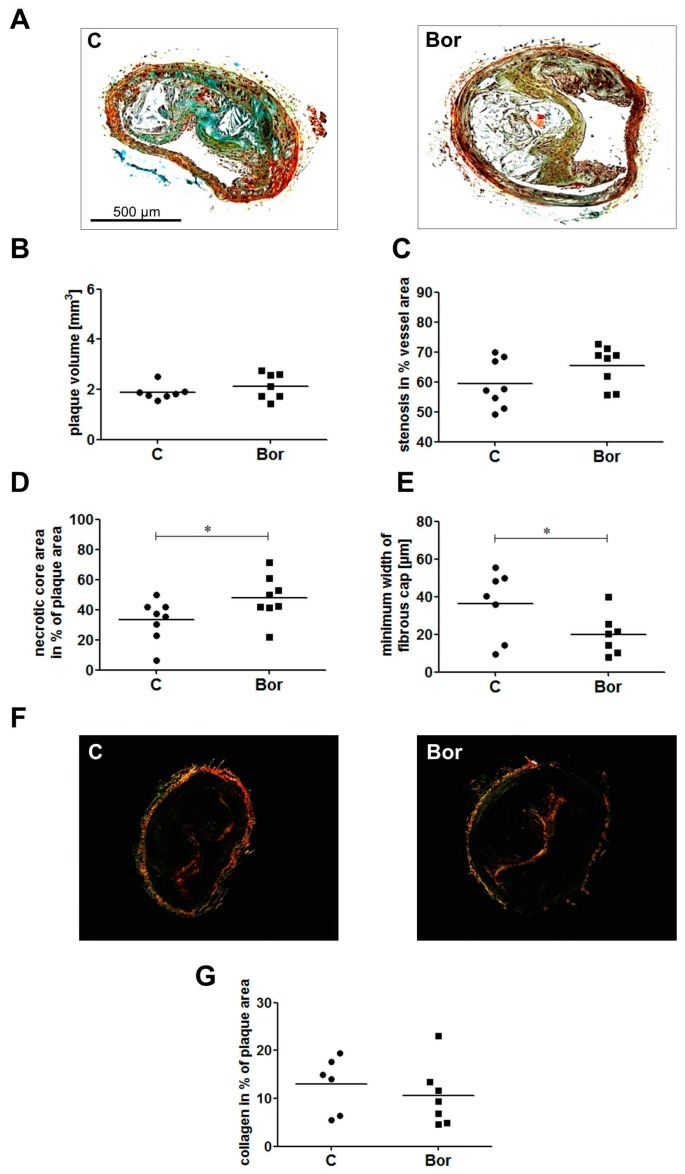
Characterization of atherosclerotic lesions in the brachiocephalic artery (BCA). (**A**) Representative serial sections of the BCA were stained with the Movat pentachrome. Plaque volume (**B**) and stenosis (**C**) were not affected by Bor treatment, confirming data from the aortic root. Bor mice showed a significantly larger necrotic core (**D**) and a significantly thinner fibrous cap (**E**). *n* = 8 per group. (**F**) Representative sections of picrosirius red-stained BCA sections were photographed under polarized light and analyzed for collagen birefringence. (**G**) Quantification of lesion collagen content. Bor treatment tended to lower collagen content of BCA lesions. *n* = 6–7 per group. Bor = bortezomib; C = saline control, * *p* < 0.05.

To gain further insight into plaque composition, we performed Movat pentachrome staining and Sirius Red-staining of paraffin sections of the brachiocephalic artery (Figure 4A,F). Plaque volume (C: 1.89 ± 0.11 mm^3^; Bor: 2.13 ± 0.19 mm^3^; *p* = 0.456; Figure 4B) and the degree of stenosis (C: 59.36% ± 2.85%; Bor: 65.41% ± 2.36%; *p* = 0.125; Figure 4C) were similar in Bor and C mice. However, lesions of Bor animals had a significantly larger necrotic core area compared to lesions of C animals (C: 33.19% ± 4.83%; Bor: 47.81% ± 5.24%; *p* = 0.029; Figure 4D). Analysis of fibrous cap width showed a significant decrease in Bor compared to C mice (C: 36.25 ± 6.74 μm; Bor: 19.86 ± 4.13 μm; *p* = 0.049; Figure 4E). Picrosirius red birefringence indicates a lower collagen content in the brachiocephalic artery (BCA) lesions of Bor mice (C: 13.00% ± 2.37%; Bor: 10.56 ± 2.42%; *p* = 0.183; Figure 4G), albeit not reaching a level of significance.

## 3. Discussion

The purpose of the current study was to investigate the influence of proteasome inhibition with bortezomib at a low dose of 50 μg/kg BW, a treatment previously shown to attenuate early atherogenesis [7], on pre-existing, advanced atherosclerosis in LDLR^−/−^ mice. Stage-dependent, opposing effects of proteasome inhibition have been hypothesized [1,8], but data from LDLR^−/−^ mice with established atherosclerosis, treated with low doses of bortezomib, were previously not available. We show that low-dose bortezomib treatment of LDLR^−/−^ mice with advanced atherosclerosis does not increase lesion burden but promotes features of plaque instability.

In the present study, we investigated the effect of low doses of bortezomib on pre-existing atherosclerosis, which was induced by feeding LDLR^−/−^ mice a Western-type diet for 18 weeks. Bortezomib had no influence on plaque burden as shown in the aorta and the BCA, but increased necrotic core size and reduced fibrous cap width in BCA plaques. Therefore, the present study illustrates the difficulty of identifying a therapeutic window for proteasome inhibition as a treatment option for atherosclerosis, but at the same time provides strong evidence for stage-dependent effects of proteasome inhibition in atherosclerosis.

Dose- and stage-dependent effects of proteasome inhibitors in atherosclerosis were discussed [8,16,17]. While dose-dependency has already been elucidated, the underlying reasons for stage-dependent effects of proteasome inhibition remain less clear. Some studies suggest that proteasomal activity is decreased in advanced atherosclerotic lesions [18,19], leading to accumulation of misfolded proteins and cellular stress [9]. Others suggest an overreactive UPS with increased proteasomal activity and enhanced nuclear factor-κB (NF-κB) activation [20]. Thus, it was hypothesized that proteasomal activity varies with disease progression. That is, proteasomal activity increases under the influence of oxidative stress in the initiation phase of atherosclerosis, possibly leading to increased NF-κB activation and an enhanced inflammatory response [1]. We provided indirect evidence of this hypothesis, since proteasome inhibition in this phase had beneficial effects [7]. Our present study fails to show beneficial effects of bortezomib on advanced atherosclerosis in LDLR^−/−^ mice. In line with the present study, van Herck and colleagues showed that a 4-week bortezomib treatment (100 μg/kg BW) of collar-induced carotid artery plaques in apolipoprotein E-deficient (ApoE^−/−^) mice promoted a rupture-prone plaque phenotype [13]. In contrast, the bortezomib dose used in our study was as low as 50 μg/kg BW and was previously shown to be beneficial in early atherosclerosis [7]. Consequently, this led to a lower degree of proteasome inhibition in the present study. However, the effects of this less pronounced proteasome inhibition were similar to those observed by van Herck et al. [13], as we observed no influence on plaque size, an increased necrotic core size and reduced collagen content. Interestingly, both studies show unaltered macrophage content of plaques after bortezomib treatment, which is underlined by the unaltered cytokine serum levels (IL-6, MCP-1) in our study. This is in contrast to our previous study showing a clear attenuation of initial macrophage infiltration by low-dose bortezomib treatment [7], apparently as a consequence of a decrease in MCP-1 and vascular cell adhesion molecule-1 (VCAM-1) expression in vascular cells [5,6,7]. In the present study, treatment was initiated in an atherosclerosis stage, where substantial lesion infiltration by macrophages had already taken place. Plaque resident macrophages are known to enhance MCP-1 levels in lesions [21], thereby promoting the recruitment of more leukocytes. We assume that this process is not sufficiently attenuated by low doses of bortezomib.

The observed promotion of plaque destabilization by bortezomib could be explained by a decreased proteasome activity in advanced atherosclerosis as recently hypothesized [1]. To further shed light on stage-specific proteasomal activity, future studies should address composition and proteolytical capacity of the proteasome in the course of atherogenesis. It is beyond the scope of the current study to answer this important question. Yet, this knowledge could enable targeted utilization of next-generation proteasome inhibitors [4] with improved specificity in atherosclerosis treatment.

Results of the present study have important clinical implications since bortezomib has become a standard therapy for multiple myeloma, especially since it is used in much higher doses to treat this malignant disease and an undefined proportion of this elderly patient cohort may exhibit coronary atherosclerosis. Indeed, recent case reports on cardiopulmonary and vascular events in multiple myeloma patients receiving proteasome inhibitor treatment [22,23] emphasize the necessity to pay particular attention to cardiac symptoms in affected patients.

In conclusion, the treatment of advanced atherosclerosis in LDLR^−/−^ mice with low doses of bortezomib promotes features of plaque instability. Taking recent studies on favorable effects of proteasome inhibition in early atherogenesis into consideration, data suggest stage-dependent effects of proteasome inhibition in atherosclerosis. Further knowledge of proteasomal composition and activity is needed to effectively explore the therapeutic potential of upcoming next-generation inhibitors.

## 4. Materials and Methods

### 4.1. Materials

Unless otherwise specified, all reagents and media were purchased from Sigma Chemicals, Germany. Bortezomib was kindly provided by Millennium Pharmaceuticals, Cambridge, MA, USA.

### 4.2. Animal Experiments

Animal experiments were approved by the local authority (G207/03, Landesamt für Gesundheit und Soziales, Berlin) and were performed according to institutional guidelines. Male 10-week-old LDLR^−/−^ mice (B6.129S7-*Ldlr^tm1Her^*/J; JAX Mice, Boston, MA, USA) were fed a high fat diet for 18 weeks ad libitum (Western-type diet containing 21% butterfat, 17% casein, 0.21% cholesterol; Ssniff, Soest, Germany). Subsequently, mice were divided into two body weight- and serum cholesterol-matched groups. A Western diet was continued for another 6 weeks plus intraperitoneal injections of bortezomib (Bor; *n* = 11) or saline (C; *n* = 11). Mice were intraperitoneally injected with 50 μg/kg BW bortezomib twice weekly. General condition and body weight were monitored. After 24 weeks, mice were fasted for two hours, anesthetized in an isoflurane-loaded box and euthanized. After perfusion with phosphate buffered saline (PBS), hearts and aortae were dissected under a stereomicroscope (Leica, Wetzlar, Germany), snap-frozen in liquid nitrogen, and stored at −80 °C or fixed in formalin.

### 4.3. Measurement of Proteasomal Activity

Measurement of proteasomal activity in liver lysates was performed as described previously [7]. Briefly, lysis of tissue was performed by grinding in liquid nitrogen, followed by repeated cycles of freezing and thawing under hypotonic conditions. After clearance of lysates by centrifugation for 20 min at 4 °C, the chymotrypsin-like activity of the proteasome was determined using the peptide substrate succinyl-Leu-Leu-Val-Tyr-(7-amino-4-methylcoumarin) (SLLVY, Bachem, Bubendorf, Switzerland) in an incubation buffer containing 225 mM Tris-HCl, pH 8.2, 45 mM KCl, 7.5 mM Mg(CH_3_COO)_2_, 7.5 mM MgCl_2_, 1.1 mM dithiothreitol, 6 mM adenosine triphosphate (ATP), 5 mM phosphocreatine, 0.2 units of phosphocreatinekinase, and 0.2 mM SLLVY. An amount of 20 μg of protein was used per assay. 7-amino-4-methylcoumarin (AMC) hydrolysis was measured after 30 min incubation at 37 °C in a GeminiEM (Molecular Devices, Sunnyvale, CA, USA) plate fluorescence reader (360 nm excitation and 460 nm emission wavelengths). The measured chymotrypsin-like (ChTL) activity in these native tissue lysates was completely blocked when lysates where incubated with 1 μM MG262 starting 30 min before the assay.

### 4.4. Staining and Analysis of Atherosclerotic Lesions

For the en face aortic lesion, analysis was performed as previously described [7]. Briefly, the dissected, formalin-fixed aortae were longitudinally opened, pinned flat on silicone gel, and stained with Oil Red O. Pinned aortae were digitally photographed. The atherosclerotic lesion area was determined using ImageJ version 1.36b software [24]. The lesion area in the aorta was calculated as the percentage of the total aortic area.

Cryosections of aortic roots were stained with Oil Red O and counterstained with hemalaun. Image analysis was performed using Zeiss Axiovision Software (Oberkochen, Germany). Results were calculated as the percentage of the lipid-stained area of the total vessel area and the plaque area.

Cross sections of formalin-fixed, paraffin-embedded brachiocephalic arteries (BCAs) were stained with the original Movat pentachrome according to the manufacturers’ protocol (Morphisto, Frankfurt am Main, Germany) and used for measurement of the necrotic core area and the width of the fibrous cap. The volume of the BCA lesion was determined as described [25].

For fibrous cap width measurements, the entire BCA was serially sectioned into 5 μm sections. One section every 75 μm was stained with the Movat pentachrome. Images were captured, and the section displaying the largest plaque area was selected. In addition, the adjacent sections of the neighboring 10 and 20 μm on both sides were stained with the Movat pentachrome, and the mean necrotic core area was calculated from these 5 sections. Accordingly, the minimum fibrous cap width was calculated.

The collagen content of BCA lesions was assessed by examination of Picrosirius Red-stained sections. Pictures were taken with identical exposure settings (AxioCam HrC, Zeiss, Oberkochen, Germany). The content of the collagen, identified by birefringence under polarized light, was quantified as the percent of plaque area for all sections. Morphometric analysis was performed on digital images using Zeiss Axiovision software.

### 4.5. Immunohistochemistry

Immunohistochemistry of acetone-fixed 5 μm aortic root cryosections was performed using anti-Mac-2 (Cedarlane Laboratories, Burlington, Ontario, Canada). Following hemalaun counterstain sections were digitally photographed under standardized conditions using Zeiss AxioCam MrC and analyzed using Zeiss AxioVision software.

### 4.6. Measurement of Serum Lipids

Plasma total cholesterol and triglyceride concentrations were measured with a colorimetric enzymatic assay (CHOD-PAP, and TG GPO-PAP (Roche-Diagnostics, Mannheim, Germany)).

### 4.7. Serum Levels of MCP-1 and IL-6

Serum levels of mouse soluble MCP-1 and mouse IL-6 were measured with a Mouse CCL2/JE/MCP-1 or IL-6 Quantikine ELISA Kit (R&D Systems Inc., Minneapolis, MN, USA) according to the manufacturer’s protocol.

### 4.8. Statistical Analysis

Data are presented as mean ± standard error of mean (SEM). Comparisons between groups were made using the Mann–Whitney or *t*-test as appropriate (GraphPad Prism Software, GraphPad, La Jolla, CA, USA). *p* < 0.05 was considered significant.

## Figures and Tables

**Table 1:** Body weight, cholesterol, and triglycerides concentrations, and proteasomal activities in LDLR^−/−^ mice.

	C (*n* = 11)	Bor (*n* = 11)
Body weight (g)	41.1 ± 1.3	42.7 ± 1.4
Total cholesterol (mg/dL)	1718 ± 154	1730 ± 130
Triglycerides (mg/dL)	1562 ± 186	1391 ± 97
Proteasomal activity in liver lysates (RFU)	1440 ± 51	1211 ± 50 *

C: control animals; Bor: animals treated with proteasome inhibitor bortezomib; LDLR^−/−^: low- density lipoprotein receptor-deficient; RFU: relative fluorescence unit. * *p* < 0.05.
